# Misconceptions Concerning Porcelain

**Published:** 1907-10-15

**Authors:** Louis Ladewich


### MISCONCEPTIONS CONCERNING PORCELAIN.

BY LOUIS LADEWICH, D.D.S.

The first misconception relates to its strength. Porcelain is stronger than it gets credit for, though this misconception is natural in view of the experience the profession has had with it. Porcelain as generally used by the profession is no stronger than its reputation indicates, but porcelain properly manipulated is one of the most serviceable materials that can be used under its proper indications.

One of its chief functions is for use in jacket or shell crowns, where if judiciously employed it is capable of a wide range of usefulness. It may be made to restore a badly broken down incisor where inlays would be treacherous, and where ordinary crowning would call for destruction of the pulp and would introduce the problem of the pin in the canal with its manifest limitations as to danger of splitting the root or perforating it. It may be used as a means of bringing into alignment many cases of irregular anterior teeth without moving the roots by properly grinding the teeth and contouring the porcelain shells so as to line them up in regular order. I have had many cases of this kind of marked improvement without destroying any pulps. One of the misconceptions about porcelain for this class of work relates to the use of facings. Facings are contraindicated in building porcelain crowns. The moment a facing is ground to fit the case its shade is changed and its strength reduced which can never be restored. More artistic and stronger work may be done by building the entire crown from the mix made by the dentist himself.

And in making the mix another misconception occurs. It is fatal to the best results and the greatest density of porcelain to jar the body into place as is usually done. When a mass of wet porcelain is jarred it causes air bubbles to settle toward the bottom of the mass, and the more it is jarred the larger are the bubbles. This may be proved by placing porcelain on the end of a spatula and jarring it. Then examine it with a glass, and it will be found full of bubbles near the bottom of the mass. Porcelain should be gently pressed or wiped into place with a spatula, in such a manner as to constitute a burnishing of the porcelain. When built up in this way it is very dense and strong and there is little shrinkage in fusing it. I seldom find it necessary to fuse a jacket crown more than once, in any tooth anterior to a molar. I use the highest fusing body I can get, and have no need for more than four shades.

A frequent use I make of the jacket crown is to employ it as a means of carrying a missing tooth. Where a single tooth has been lost in the arch I grind one of the adjacent teeth and make a jacket crown for it. I then trephine the process where the tooth was lost and make a porcelain tooth—crown and root—to fit into the vacant space. The two are fused together with porcelain and adjusted to position—the jacket cemented and the attached tooth implanted. Some of these cases have been in the mouth for more than a year and are still doing good service.

In grinding natural teeth for jacket crowns I invariably keep them dry, directing a jet of compressed air immediately at the point where the stone or bur touches the tooth. By this means I am usually able to grind the most sensitive tooth with little discomfort. But there must be no hesitation or misdirected manipulation. The operator must know in advance precisely the form he wishes to give the tooth, and then go directly to that form. It should never take more than ten, or at least fifteen minutes, to grind down any tooth for a jacket crown. In truing up the walls and making a shoulder at the gingival margin 1 use sharp fissure burs. And they must be sharp. The moment a stone is not true or a bur is the least dull it is discarded. I have used as many as six burs in preparing a tooth for a crown.

The gauge of platinum used for the matrix is 1-1000 of an inch, and it is simply wrapped around the tooth and fitted without soldering. In this way it can be easily peeled from the inside of the crown after fusing. The platinum used should be fresh from the manufacturer and not subjected to the slightest manipulation previous to being adjusted to the tooth. I buy mine by the ounce to be assured that it is not handled by the dealer in any way. Platinum under these conditions is almost as adaptable and soft as tin foil, but it is quickly hardened and stiffened by manipulation.

The possibilities of porcelain in this work are well nigh unlimited, and if any of the statements herein made seem radical or unreasonable I can only add that they are capable of demonstration at any time. Porcelain inlays have been heralded throughout the world, but porcelain jacket crowns are capable of a far wider range of usefulness than inlays—*Dental Review.*

